# Modern Approaches to Diagnosis and Evaluation of Survival Prognosis in Patients with Pancreatic Cancer

**DOI:** 10.3390/ijms27135867

**Published:** 2026-06-29

**Authors:** Maria Getsina, Nikolay Tsyba, Ekaterina Chernevskaya

**Affiliations:** Federal Research and Clinical Center of Intensive Care Medicine and Rehabilitology, 25-2 Petrovka Str., 107031 Moscow, Russia; tsyba77@gmail.com (N.T.); echernevskaya@fnkcrr.ru (E.C.)

**Keywords:** pancreatic cancer, microbiome, metabolites, metagenomic changes, diagnostic markers

## Abstract

Pancreatic cancer is among the most aggressive malignancies, and late diagnosis remains a key challenge. For a systematic review of pancreatic cancer diagnosis and prognosis, Scopus and Web of Science databases were used for the period from 2016 to 2026. The search query included the following keywords and their combinations: pancreatic cancer, diagnosis, early detection, prognosis, biomarkers, metabolomic profiling, CA19-9, microbiome, metagenomic changes, circulating tumor DNA, genomic analysis. Inclusion criteria included only articles published in English. Exclusion criteria included case reports and studies that did not examine pancreatic cancer. Our analysis demonstrates that integrating multi-omics data, particularly combining traditional CA19-9 with circulating tumor DNA (ctDNA) and metabolomic profiles (lipids, amino acids, carbohydrates), significantly improves diagnostic accuracy. Microbiome composition and genomic alterations further refine risk stratification and prognostic assessment. The synergistic use of these biomarkers may facilitate the development of screening, early diagnosis, risk stratification, and treatment optimization. However, the introduction of new diagnostic approaches into clinical practice requires additional verification, standardization and prospective clinical studies.

## 1. Introduction

Pancreatic cancer is among the most aggressive malignancies. Despite advances in clinical care and a deeper understanding of the disease’s biology, five-year survival remains low—less than 9% [1,2,3]. Since pancreatic ductal adenocarcinoma (PDAC) accounts for 95% of pancreatic cancer cases, the obtained statistical data can be extrapolated to pancreatic cancer in general. Global trends indicate an increase in the incidence of PDAC, although prevalence rates vary significantly by region. In countries with a low human incidence index, the incidence rate is approximately five times higher than in North America: standard rates are 8.5 cases per 100,000 people in economically developed Europe, 8.0 in North America, and only 1.3 in Southeast Asia [4]. The incidence rate of PDAC in Russia was approximately 6.8 per 100,000 people [5].

PDAC incidence can increase at any age, but peak incidence occurs between 60 and 80 years of age. Late diagnosis remains a key problem in PDAC: on average, approximately 10 years pass from the onset of mutation to the formation of the primary tumor, and another 10 years are required for the disease to progress to death [6,7]. However, this timeline represents a generalized approximation; substantial variability exists across patients due to differences in molecular pathogenesis, host factors, and environmental influences. Therefore, these estimates should be interpreted with caution.

Currently, treatment methods remain chemotherapy and surgery. Still, their effectiveness is limited by several factors: 80–85% of patients are already inoperable at the time of diagnosis, and there is a high risk of postoperative complications that negatively impact survival [8,9].

Recent advances in metabolomics open new perspectives for solving problems in the diagnosis and treatment of PDAC. Metabolomic profiling reflects the dynamic state of biological processes and can serve as an indicator of premature pathological changes [10].

Studies show that PDAC invariably involves severe disturbances in metabolic pathways. Intratumor microbiomes and altered metabolic networks, including microbiota-derived metabolites, play a crucial role in the progression of PDAC [10,11].

Gut microbiota metabolites have received particular attention: microbiomes have been shown to influence carcinogenesis through a number of factors, including modulation of the immune response, production of metabolites with pro- or anticarcinogenic properties (such as short-chain fatty acids and secondary bile acids), and effects on inflammatory processes and intestinal barrier permeability.

Preliminary studies have identified characteristic metabolomic signatures that can differentiate patients with PDAC from healthy individuals and patients with pancreatic ductal diseases. In particular, in patients with PDAC, measurement of certain fatty acids and acylcarnitines reduces the levels of certain amino acids (e.g., tryptophan) and various microbiota-associated metabolite profiles (including indoles and phenolic compounds) [12].

The use of metabolomic markers in personalized medicine for PDAC may offer several advantages: early diagnosis of the disease at a pre-symptomatic stage; prognosis of the disease course by identifying molecular subtypes with different prognoses; personalization of therapy by selecting the most effective treatment regimen based on the patient’s metabolomic profile; and monitoring of treatment response and early relapse.

This review aims to fill the gap between basic research on metabolic pathways and the clinical application of this knowledge, offering a systematization of approaches to the diagnosis, prognosis and evaluation of treatment efficacy in PDAC.

## 2. Diagnostic Methods

**Figure 1:**
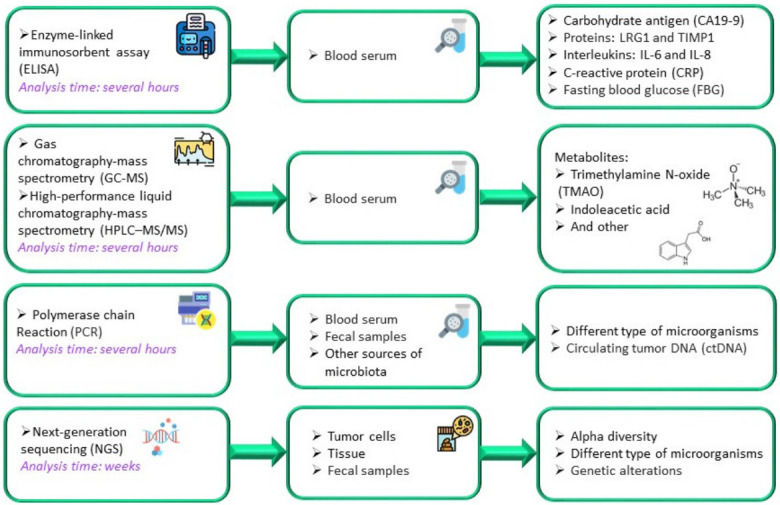
Modern methods of analysis that allow us to evaluate changes in the composition of biological fluids and tissues in the prognosis, treatment and diagnosis of pancreatic cancer.

Current Russian guidelines for laboratory diagnostics of PDAC are rated as recommendation Grade C and evidence level 5, reflecting a lack of high-quality clinical studies or consensus regarding the effectiveness of the recommended laboratory tests. Despite these limitations, several laboratory methods show potential to improve diagnosis and treatment. Figure 1 shows the potential laboratory research possibilities for the diagnosis and analysis of PDAC using various methods of biomaterial analysis.

Modern methods of analysis allow us to evaluate changes in the composition of biological fluids and tissues in the prognosis, treatment and diagnosis of PDAC.

The key diagnostic method remains enzyme-linked immunosorbent assay (ELISA), used to determine the level of carbohydrate antigen 19-9 (CA19-9), a recognized circulating biomarker of pancreatic cancer [13,14,15]. CA19-9 is primarily used for monitoring an already established diagnosis (assessment of response to therapy, detection of relapses), not for primary screening.

Molecular and metabolomic methods are promising areas for the development of laboratory diagnostics for PDAC. Among the molecular approaches are polymerase chain reaction (PCR), used to study genetic polymorphisms and circulating tumor DNA (ctDNA) [16,17]; sequencing techniques, including single-cell RNA sequencing (scRNA-seq), which enable the direct assessment of changes in tumor cells and facilitate microbiome analysis [10,11,18,19,20]; and microbiome analysis, which serves as a source of potential non-invasive biomarkers based on the composition of oral and intestinal microbiota [21,22,23,24].

The microbiome does not act in isolation: its metabolic activity directly influences host physiology and may contribute to carcinogenesis. This functional link underscores the value of metabolomic analysis, which captures the biochemical output of both human cells and microbial communities. In parallel, metabolomic analysis using gas chromatography–mass spectrometry (GC-MS) and high-performance liquid chromatography (HPLC) enables the identification of characteristic “metabolic signatures” in blood serum (changes in amino acid, lipid, and bile acid profiles), which can occur long before the appearance of a clinically visible tumor [13,25,26,27,28].

Microbiome analysis is emerging as a promising approach for the early diagnosis of PDAC. Changes in the composition of the oral, fecal, and pancreatic microbiomes have been linked to an increased risk of PDAC [21]. Specifically, differences between the oral and intestinal microbiomes correlate with a higher risk of PDAC, positioning them as potential non-invasive biomarkers. For instance, patients with PDAC exhibit reduced gut microbiota diversity, a decreased proportion of Firmicutes, and an elevated abundance of pathogenic and lipopolysaccharide (LPS)-producing Proteobacteria, such as *Klebsiella pneumoniae* and *Enterobacteriaceae* [22]. Additionally, dysbiosis of the tongue plaque microbiota has been observed in patients with primary pancreatic cancer, characterized by an overrepresentation of *Leptotrichia*, *Fusobacterium*, *Rothia*, and other taxa [24]. Furthermore, alterations in the salivary microbiota are associated not only with pancreatic cancer but also with chronic pancreatitis [23].

The choice of biospecimen is determined by the specific goals of the study. Blood or urine samples provide insights into systemic processes, including inflammation, immune responses, and the functional status of vital organs. Tissue or pancreatic juice samples enabling the detection of tumor-specific metabolites (oncometabolites), key gene mutations (such as KRAS and TP53), and the composition of infiltrating immune cells [29]. Fecal samples are primarily used for comprehensive microbiota analysis, which can reveal associations between gut microbial composition and disease states. Moreover, the intestine is the body’s largest “input filter,” and its dysbiosis can trigger systemic inflammation and immune dysfunction, contributing to carcinogenesis [21,30].

Integrative multi-omics analysis enhances diagnostic value by combining data from different sample types and methods, providing a more comprehensive understanding of disease mechanisms.

## 3. Metabolic Profile in Pancreatic Cancer

Metabolites, as direct readouts of cellular metabolism, can reflect disease-associated changes long before structural or genetic abnormalities become apparent. The following section explores how metabolomic signatures across various biospecimens may serve as sensitive and non-invasive biomarkers for early pancreatic cancer diagnosis.

### 3.1. Metabolic Alterations as Diagnostic and Prognostic Tools in Pancreatic Cancer

Metabolic reorganization observed in most tumor cells is a fundamental characteristic of cancer [31].

Table 1 clearly demonstrates the wide range of metabolites used in the diagnosis and prognosis of pancreatic cancer. These include carbohydrates and related compounds (glucose, xylitol, 1,5-anhydro-D-glucitol, glycated hemoglobin (HbA1c)), amino acids and their derivatives (histidine, indole-3-acetic acid, 5-hydroxytryptophan, N-succinyl-L-diaminopimelic acid), lipids and their derivatives (palmitic acid, cholesterol, ultra-long-chain fatty acids, phosphatidylcholines, sphingomyelins, oleoyl-L-carnitine, phosphatidylethanolamine), organic acids (including lactate and other metabolic intermediates), energy and signaling molecules (insulin, heat shock protein 70 (HSP70)), and phospholipid and other metabolites with diagnostic significance (e.g., PC.aa.C38:4, PC.ae.C42:5, PC.ae.C38:6) [12,26,32,33,34].

Data analysis demonstrates the diversity of metabolic markers and detection methods used in pancreatic cancer diagnostics. The most effective panels combine multiple metabolite types, such as lipids, amino acids, and carbohydrates, thereby increasing diagnostic sensitivity and specificity when compared with individual markers.

The combination of palmitic acid with CA19-9 allows to achieve an ideal AUC of 1.000 [12], Multi-component metabolomic panels also demonstrate high performance: a panel of 76 metabolites provides an AUC of 0.9773 for differential diagnosis [33], and a two-metabolite panel (N-succinyl-L-diaminopimelic acid and phosphatidylethanolamine) an AUC = 0.951 with a sensitivity of 93.3% and specificity of 93.1%, which is especially valuable for screening PDAC in patients with diabetes [42]. Individual metabolites such as 1,5-anhydro-D-glucitol (AUC = 0.83499, sensitivity 86%, specificity 71.4%) [26] and the combination of CA19-9 with HbA1c (AUC = 0.81, sensitivity 70%, specificity 96.5%) [14] also show good results. A panel of serum cytokines (B7-1/CD80, EG-VEGF/PK1, IL-29, etc.) complements the diagnostic potential, providing an accuracy of 92.3%, sensitivity 85.57% and specificity of 100% [40], which is significantly superior to the performance of CA19-9 alone. Immune-inflammatory markers (IL-6/IL-8, LNR, NLR, PLR, CRP) provide prognostic information on survival and response to therapy that is not available using traditional biochemical tests. Furthermore, integrated models, such as the SEER-based nomogram (AUC = 0.845 for predicting lymph node metastasis) [37], expand the possibilities for risk stratification and treatment personalization. Thus, comprehensive panels combining metabolites, immunological parameters, and clinical indices can significantly improve the accuracy of PDAC diagnostics (including at early stages), improve differential diagnostics, obtain prognostic data for therapy selection, and take into account the clinical context (diabetes, inflammation, postoperative status), making them a promising basis for the next generation of diagnostic and prognostic tools in PDAC management.

Notably, metabolic factors themselves are closely linked to PDAC risk. A meta-analysis revealed a strong association between metabolic syndrome and PDAC: individuals with metabolic syndrome face a higher risk of developing the disease, regardless of gender [43]. This link is largely explained by hypertension, hyperglycemia, and low HDL cholesterol levels, while the prevalence of PDAC appears independent of obesity and hypertriglyceridemia. Patients with diabetes have a significantly increased risk of developing PDAC. A positive correlation between blood glucose levels and CA19-9 levels was found in people with diabetes without malignancies, as well as in patients with pancreatic neuroendocrine tumors [15]. Furthermore, a link between CA19-9 levels and glycated hemoglobin (HbA1c) levels was found [14].

CA19-9 is an established biomarker for PDAC, allowing for the differentiation of resectable tumors from chronic pancreatitis. However, its value for early diagnosis is limited. The combination of CA19-9 with leucine-rich alpha-2-glycoprotein 1 (LRG1) and tissue inhibitor of metalloproteinases-1 (TIMP1) increases sensitivity by 13.2% while maintaining high specificity (99%) for identifying cases diagnosed within a year of blood sampling (*p* = 0.031) [13]. The cutoff level of CA19-9 for non-diabetic patients is 37 U/mL.

Several studies demonstrate that tumors behave differently depending on their anatomical location. Identifying the pancreatic metabolic response to pathophysiological stimuli is essential for disease assessment. An integrated analysis identified three highly enriched metabolic pathways: branched-chain amino acid (BCAA) biosynthesis, glycerophospholipid metabolism, and phenylalanine metabolism. In a cell line, BCAAs promoted pancreatic cancer proliferation and inhibited oxaliplatin-induced apoptosis. The integrated analysis identifies key metabolites and enzyme-encoded genes between pancreatic head and body/tail adenocarcinoma. Therapies targeting BCAA metabolism may be promising, particularly in pancreatic body/tail cancer [20].

Despite these advances, several challenges remain before metabolomic analysis results can be implemented into routine clinical practice. These include variability in metabolic profiles across disease stages and comorbidities (e.g., diabetes), the need to validate biomarkers in large patient cohorts, and the standardization of analytical protocols (e.g., HPLC, LC-MS/MS) to ensure reproducible results. Addressing these challenges is essential for translating scientific advances into clinical practice.

### 3.2. Mitochondrial Dysfunction and Metabolic Reprogramming in Pancreatic Cancer

Cancer can generally be viewed as a “selfish” metabolic disease, in which the tumor reprograms both its own metabolism and that of surrounding cells. The metabolic reorganization observed in cancer represents a source of potential diagnostic markers. A hallmark of cancer metabolism is the Warburg effect, aerobic glycolysis, in which tumor cells consume more glucose and convert it to lactate even in the presence of oxygen [29,44,45]. In PDAC, this is accompanied by complex reprogramming of glucose, amino acid, and lipid metabolism [46], leading to various metabolic phenotypes (Warburg, reverse Warburg, lipid-dependent, glutaminolytic) determined by genetic mutations and stromal signals [47,48]. Mitochondrial dysfunction in cancer includes genetic, molecular, and biochemical alterations, including mitochondrial DNA (mtDNA) changes (point mutations, polymorphisms), which can influence the risk of cancer development and its progression [16,49]. Key metabolic shifts include restructuring of amino acid metabolism and dysregulation of the tricarboxylic acid cycle (TCA cycle). Notably, intermediates such as succinate and fumarate act as oncometabolites: accumulation of succinate inhibits α-ketoglutarate-dependent dioxygenases, causing epigenetic changes and promoting tumor progression [50,51]. These TCA cycle intermediates also have non-metabolic signaling functions in immune and tumor cells [25], making them potential biomarkers for early cancer diagnosis [51]. At the same time, patients were found to have decreased serum levels of fumaric and succinic acids, which, at first glance, seems at odds with the previously mentioned accumulation of citric acid cycle intermediates as oncometabolites [52]. However, this discrepancy can be explained by several mechanisms: the accumulation of these metabolites directly in tissues (as opposed to systemic depletion), the action of compensatory mechanisms aimed at normalizing metabolic balance, their increased utilization in alternative metabolic pathways, or altered renal clearance or tissue distribution. Taken together, these mechanisms highlight the complexity of metabolic reprogramming in pancreatic cancer and indicate the need for comprehensive, multicomponent approaches to biomarker development. Metabolic reprogramming in pancreatic cancer is associated with the activation of oncogenic signaling pathways (including PI3K/Akt/mTORC1, Ras/Raf/MEK/ERK) [29,53], which enhance glucose transport and glycolysis due to increased expression of glucose and lactate transporters (GLUT, MCT4) and activation of glycolytic enzymes (HK2, PFK1, ENO1, PKM2, LDHA). Simultaneously, there is suppression of protective factors, such as tumor suppressors (p53, PTEN) and metabolic regulators (sirtuins 3/6, AMPK), which weakens normal control over cell growth and stress response and promotes tumor survival and progression. Furthermore, dysregulation of mitochondrial factors (Bcl 2, HIF, NRF2, etc.) leads to global metabolic changes [54,55,56,57], and mutations in citric acid cycle enzymes (isocitrate dehydrogenase, succinate dehydrogenase, fumarate hydratase) further disrupt metabolite regulation and stimulate carcinogenesis [50].

These data on mitochondrial and metabolic changes provide a compelling rationale for developing metabolomic diagnostic approaches for pancreatic cancer. Serum metabolites, including citric acid cycle intermediates, amino acids, lipids, and other oncometabolites, represent promising targets for biomarker panels. For example, elevated lactate-to-glucose ratio in serum reflects the Warburg effect and correlates with tumor burden. Combining metabolomic data with other biomarkers (e.g., inflammatory factors such as IL-6 and CRP, or ctDNA) may improve diagnostic and prognostic accuracy [38,39,40,41], allowing for more personalized survival assessment and treatment strategies.

## 4. Microbiome as a Tool for Diagnostics and Prognosis

### 4.1. Taxonomy of Microbiota as a Risk Factor for the Development and Diagnostic Marker of Pancreatic Cancer

The composition of the microbiota is associated with the risk of developing pancreatic cancer and can serve as a basis for non-invasive diagnostic methods. Analysis of the presented data demonstrates a clear link between the composition of the microbiota at various body sites and the development of PDAC, as well as the prognosis of the disease; see Table 2.

Microbiome biomarkers represent a promising tool for noninvasive and minimally invasive diagnosis of pancreatic cancer, demonstrating clinically significant sensitivity and specificity, in some cases comparable to or even superior to traditional tests. For example, a combination of two microbial biomarkers in saliva (*Neisseria elongata* and *Streptococcus mitis*) provides an AUC of 0.90 with a sensitivity of 96.4% and a specificity of 82.1% [23], which is significantly higher than the typical values of CA19-9 alone (mean AUC values for CA19-9 typically range from 0.75 to 0.85, with a sensitivity of approximately 70–80% and a specificity of 80–90%) [14]. The combination of microbial and traditional markers increases the AUC from 0.84 to 0.94 [58]. A similar effect is observed with expanded panels of microbial markers. For example, the inclusion of Bacillus clausii in a panel of three tumor tissue taxa (*Pseudoxanthomonas*, *Saccharopolyspora*, *Streptomyces*) increases the AUC from 88.89% to 97.51% [65]. The advantages of the microbiome approach also include the ability to use a variety of sample types (feces, saliva, duodenal fluid, tumor tissue), which expands diagnostic options, including for patients for whom invasive procedures are contraindicated.

The data obtained not only confirm the link between the microbiota and the development and prognosis of PDAC but also reveal potential mechanisms for this interaction. For example, a shift in the *Bacteroidetes*/*Firmicutes* ratio may reflect altered metabolic functions of the microbiome, in particular, a decrease in the production of short-chain fatty acids (SCFAs), which have anti-inflammatory and antitumor effects [61]. Enrichment with taxa such as *Prevotella* and *Veillonella* is likely associated with increased fermentation processes and changes in local pH, which can influence intestinal barrier permeability and systemic inflammation [58,59].

Notably, the link between the oral microbiota and PDAC may be mediated by chronic inflammation: *Porphyromonas gingivalis*, which is associated with an increased risk of cancer, is known to induce proinflammatory cytokines and activate signaling pathways involved in carcinogenesis. Conversely, the protective effect of *Fusobacteria* and *Leptotrichia* may be due to competition with pathogens or modulation of the immune response [64].

The tumor and duodenal fluid microbiome appear to actively interact with the tumor microenvironment. High alpha diversity, characteristic of patients with long-term survival, may facilitate more effective CD8+ T-cell infiltration and inhibit tumor growth. The identified signature profile (*Pseudoxanthomonas*/*Streptomyces*/*Saccharopolyspora*/*Bacillus clausii*) may influence local immunity—for example, by producing antimicrobial peptides or metabolites that modulate immune cell activity [65].

Furthermore, the influence of treatment on the microbiota’s composition highlights its dynamism and dependence on external factors. For example, differences in the microbiome between groups receiving different infusion therapy regimens [59] suggest that standard treatment protocols may unintentionally alter the microbial balance, either favor beneficial commensals or promote the proliferation of opportunistic species. This raises the question of the need to consider the microbiome profile when planning treatment to minimize negative consequences and improve therapeutic efficacy.

Experimental studies in mouse models support the possibility of a direct influence of intestinal bacteria on the pancreas [66]. When fluorescently labeled *Enterococcus faecalis* was administered via oral gavage, the bacteria migrated to the pancreas, demonstrating a potential pathway for microbes to enter the organ and impact the microenvironment. In healthy control groups, the intestinal microbiota is typically dominated by *Bacteroidetes* and *Firmicutes*, while *Actinobacteria*, *Proteobacteria*, and *Verrucomicrobia* make up a minor proportion. In contrast, patients with PDAC exhibit elevated levels of *Proteobacteria*, *Actinobacteria*, *Fusobacteria*, and *Verrucomicrobia.* Notably, *Proteobacteria* are also enriched in the intrapancreatic microbiome and are associated with disease progression [66].

Beyond specific bacterial translocation, broader epidemiological patterns have also been explored. For instance, epidemiological data suggest that *Helicobacter pylori* (*H. pylori*) may be a contributing factor to pancreatic cancer. A meta-analysis of 17 studies involving 67,910 individuals, including 64,372 controls and 3538 patients with pancreatic cancer, found no significant association between *H. pylori* infection and the risk of pancreatic cancer [1]. Overall, *H. pylori* infection does not significantly increase the risk of pancreatic cancer. Furthermore, *H. pylori* should be considered within the broader context of non-hereditary risk factors for PDAC include age (>55 years), chronic pancreatitis, diabetes, tobacco smoking, obesity, alcohol abuse, dietary factors, and exposure to toxins [67]. This multifactorial landscape implies that the carcinogenic potential of cytotoxin-associated protein A (CagA+) *H. pylori* may be most pronounced in individuals who already have one or more of these risk conditions [1]. For example, chronic inflammation from pancreatitis or metabolic disturbances in diabetes could create a permissive microenvironment where the pro-inflammatory and genotoxic effects of CagA+ strains become particularly harmful. Thus, while *H. pylori* alone may not be a major risk factor, its virulent strains could act synergistically with other established risk factors to increase PDAC susceptibility.

The relationship between microbes and immunity in the development of PDAC is dual [68]. On the one hand, the microbiota can enhance antitumor immunity, while on the other, it can exert a protumor effect. For example, changes in the composition of the microbiota in PDAC have been described: an enrichment of *Klebsiella* spp. and *Bacteroides* is combined with a decrease in the abundance of beneficial and potentially anti-inflammatory strains, such as *Faecalibacterium prausnitzii* and *Roseburia* spp. [69]. The results of a two-sample Mendelian randomized analysis using genome-wide association study data confirmed a causal relationship between gut microbiota composition and PDAC risk: *Senegalimassilia* exerts a protective effect against PDAC, while *Odoribacter*, *Ruminiclostridium 9*, *Ruminococcaceae* (UCG011) and *Streptococcus* act as causal factors in its development [57].

This highlights the importance of microbial community balance: dysbiosis can create conditions conducive to carcinogenesis, while restoration of the normal microbiota composition has the potential to improve prognosis and enhance therapeutic efficacy.

However, a number of methodological limitations must be considered when interpreting these results. The choice of sequencing method, 16S rRNA sequencing (used in most studies, e.g., [59,60,61,62,63,65]) or shotgun metagenomics (e.g., [19,58]), can influence the results obtained and, consequently, bias the interpretation. For example, 16S NGS provides taxonomic information at the genus or family level, it does not provide functional data, may miss low-abundance taxa, and cannot detect viral or fungal components; in contrast, metagenomics offers species-level resolution and functional profiling (e.g., metabolic pathways), but is more expensive and computationally intensive. Batch effects and technical variability further complicate comparisons of results across studies: differences in DNA extraction protocols, sequencing platforms and bioinformatics pipelines, and normalization methods (rarefaction, relative abundance) can distort true biological signals, especially in low-biomass samples (e.g., duodenal fluid [62]). Factors such as diet (short-term changes in diet can rapidly alter the composition of the gut microbiota), comorbidities (chronic pancreatitis, diabetes, and obesity independently affect the microbiome), medications (antibiotics, proton pump inhibitors, and chemotherapy significantly alter microbial communities), and lifestyle factors (smoking and alcohol consumption) can mask disease-specific signatures or bias biomarker searches. Heterogeneity of sample types and locations also poses challenges: there are discrepancies between microbiome profiles from fecal, duodenal, oral, and intratumoral samples and low microbial biomass in some samples increases the risk of contamination during DNA extraction. Finally, statistical and analytical challenges arise from the high dimensionality of microbiome data (thousands of taxa compared to limited sample sizes), which increases the risk of false positives, and the lack of standardized thresholds for α-diversity or taxonomic thresholds, which complicates clinical interpretation.

Thus, while the microbiota holds promise as a biomarker for PDAC, rigorous methodological considerations are essential for translating discoveries into clinical practice. Despite these limitations, the microbiota not only serves as a biomarker for PDAC but is also actively involved in its pathogenesis through metabolic, immune, and inflammatory mechanisms, offering promise for the development of microbiome-based strategies for the diagnosis, prevention, and treatment of PDAC.

### 4.2. Microbiota Metabolites: Diagnostic and Therapeutic Potential

The functional state of the microbiota, in addition to its structural composition, plays a critical role in host physiology and pathology. Microbial metabolites are key regulators of mitochondrial function, immune responses, and cancer progression, thereby serving as valuable diagnostic and prognostic biomarkers and significantly influencing treatment efficacy.

A range of microbial metabolites, including bile acids, tryptophan derivatives, SCFAs, and components involved in the TCA cycle, are known to modulate mitochondrial activity. These metabolites contribute to maintaining normal gastrointestinal homeostasis, while their dysregulation is implicated in gastrointestinal diseases and carcinogenesis [44]. For instance, SCFAs (e.g., butyrate, propionate, and acetate) exert anti-inflammatory effects and support epithelial integrity, whereas imbalances in bile acid metabolism can promote inflammation and DNA damage, potentially driving oncogenic processes. A three-marker panel of microbial-associated metabolites was developed to estimate the 5-year risk of developing PDAC. A total of 14 microbial metabolites were detected and quantified, including nine indole derivatives, two secondary bile acids, 5-hydroxytryptophan, acetylcadaverine, and trimethylamine N-oxide (TMAO). The authors note that elevated levels of TMAO and indoleacrylic acid were associated with several bacterial phyla, including *Bacillota*, *Bacteroidota*, *Actinomycetota*, and others which are closely related to PDAC [28].

The relationship between TMAO and PDAC remains a subject of active research, with the focus gradually shifting from identifying risk factors to finding new therapeutic approaches. Elevated TMAO levels are associated with several bacterial phyla, including *Bacillota*, *Bacteroidota*, *Actinomyceta*, and *Pseudomonadota*. Notably, TMAO enhances antitumor immunity in PDAC: experimental data in mouse models show that both intraperitoneal administration of TMAO and dietary choline supplementation reduce tumor growth—this effect is mediated by stimulation of tumor-associated macrophages and activation of effector T-cell responses [70]. Furthermore, levels of bacteria that contain CutC (the enzyme responsible for producing the trimethylamine (TMA) precursor) correlate with long-term survival in patients with PDAC.

Although large epidemiological studies directly linking TMAO to PDAC are not yet available, a number of lines of evidence support the hypothesis of its potential role. At the molecular level, TMAO induces inflammation (via activation of the NLRP3 inflammasome), oxidative stress, and DNA damage, processes directly involved in pancreatic carcinogenesis [71,72]. TMA serves as a substrate for the formation of N-nitroso compounds, proven pancreatic carcinogens [73,74]. Alterations in the composition of the gut microbiota, including TMA-producing microbes, are observed in PDAC, and intratumoral bacteria modulate the tumor microenvironment [75,76]. Also, TMAO has demonstrated prognostic value in colorectal cancer [77], suggesting its potential applicability in the diagnosis/prognosis of PDAC. TMAO’s role may be dual: some studies point to its protective functions as an osmolyte and protein stabilizer. TMAO levels depend on multiple factors, including diet, microbiota composition, intestinal barrier permeability, liver enzyme activity, and renal excretion rate [78], so interpreting it as a biomarker requires a comprehensive approach. Of particular interest are studies examining TMAO in combination with other biomarkers (e.g., CA19-9 and microbiota composition)—this may improve the accuracy of early PDAC diagnosis and improve prognosis stratification in patients.

Research attention has also focused on indole-3-acetic acid (3-IAA), a tryptophan derivative with clinical significance. Recent studies have revealed a multifaceted relationship between 3-IAA and pancreatic cancer: its role as a signaling molecule via the aryl hydrocarbon receptor (AhR) [79,80], its potential as an antitumor agent, and its significance as a microbiome-derived biomarker for the diagnosis of PDAC.

3-IAA levels are significantly elevated in patients responding to treatment. In two independent cohorts of patients with pancreatic cancer, a robust correlation was observed between 3-IAA levels and chemotherapy efficacy. This finding strongly suggests that 3-IAA has significant potential as a prognostic marker of treatment response [27]. Direct measurements of 3-IAA levels in human cancer tissues have also provided important insights. A study using gas chromatography/high-resolution ion-selective mass spectrometry (GRIS) revealed that 3-IAA levels were significantly elevated in esophageal squamous cell carcinoma tissues (1400–4700 ng/g fresh weight) compared with adjacent normal tissues (90–390 ng/g fresh weight) [81]. This corresponds to a 5- to 10-fold increase in 3-IAA content in tumor tissues. The authors suggested that increased 3-IAA production may be associated with cell proliferation in early stages of squamous cell carcinoma [81]. Although the study focused on esophageal cancer, it raises the question of whether 3-IAA accumulation may be a characteristic feature of other epithelial malignancies, including pancreatic cancer, thus requiring further study.

The interaction between the kynurenine and indole pathways is particularly important for PDAC. Indoleamine 2,3-dioxygenase 1 (IDO1) expression is elevated in many solid tumors, including prostate cancer, and correlates with an unfavorable prognosis [82]. The IDO1/TDO-Kyn-AhR signaling axis promotes tumor immune evasion through multiple mechanisms, including inhibition of antitumor immune responses [83]. Therefore, strategies for restoring a healthy balance between the kynurenine and indole pathways are being explored, for example, through stimulation of bacteria producing 3-IAA, the use of IDO1 inhibitors, and the administration of direct AhR modulators. These approaches are being considered as potential therapeutic strategies.

The role of LPS, which are derived from Gram-negative bacteria, deserves special consideration. These molecules play a significant role in the development of chronic inflammation and carcinogenesis. Clinical data show that patients with chronic pancreatitis have higher LPS levels. Importantly, these elevated levels correlate with disease duration and may impair glucose metabolism. Consequently, such metabolic disturbances may increase the risk of pancreatic cancer [66,84].

Thus, microbial metabolites, including TMAO, 3-IAA, and LPS, have multifaceted effects on tumor progression and treatment response. Given their proven association with clinical outcomes, these compounds hold significant potential both diagnostic markers and therapeutic targets. In particular, they may aid in the development of dietary strategies and approaches to modulating the microbiome to improve pancreatic cancer treatment.

One possible mechanism is that a healthy microbiota, under homeostatic conditions, supports mitochondrial bioenergetics and cellular signaling mechanisms through the supply of metabolites, whereas dysbiosis in chronic inflammation and prostate cancer creates a proinflammatory environment by promoting aerobic glycolysis, inducing oxidative phosphorylation and fatty acid neutralization, impairing mitochondrial permeability, and blocking apoptosis [44]. Dysbiotic microbiota at various levels (intestine, pancreas, oral cavity) directly influences the microenvironment in pancreatic cancer, disrupting metabolism and promoting carcinogenesis.

In summary, the microbiome holds significant potential for improving pancreatic cancer treatment. Noninvasive methods based on stool, oral, or duodenal microbiome profiles can complement traditional markers such as CA19-9. The microbiome also offers the potential to improve prognosis: tumor microbiome signatures can predict patient survival and aid in treatment decisions. Optimization of therapy, which can be achieved through microbiome modulation [65,85], also holds promise. The intestinal microbiota is involved in the metabolism of chemotherapeutic agents and the formation of the tumor microenvironment, determining the effectiveness of both traditional chemotherapy and immunotherapy in pancreatic cancer through drug-induced changes in the microbial composition. Dietary interventions targeting specific microbial metabolites, such as TMAO or 3-IAA, may enhance the effectiveness of chemotherapy.

However, several challenges remain. First, the effectiveness of microbiome biomarkers needs to be validated across large, diverse patient cohorts. Secondly, standardization of sample collection and analysis protocols is essential, particularly when comparing different methodologies, such as metagenomics and 16S rRNA sequencing. Thirdly, it is crucial to understand better the influence of co-factors, including diet, medications, and comorbidities, on microbiome profiles. Finally, the development of microbiome-targeted therapies and their successful integration into clinical practice remain key challenges for future research.

## 5. Molecular Genetic and Cellular Markers

Recent studies have significantly advanced our understanding of molecular and cellular markers in pancreatic cancer, providing new insights into prognosis and potential therapeutic targets.

Table 3 presents studies on metagenomic changes occurring directly in pancreatic tissues, reflecting alterations in tumor cells and healthy tissues, as well as the potential for diagnosis or prognosis based on these changes. Current studies using scRNA-seq, genomic analysis, and metabolomics reveal the heterogeneity of PDAC cells, including immune profiles, microbiome changes, and metabolic pathways. Comprehensive analysis reveals consistent restructuring of the microbiome, metabolome, and genome during prostate cancer progression from squamous cell carcinoma to metastasis.

Modern molecular genetic and transcriptomic methods (NGS, scRNA-seq, ddPCR, etc.) are actively used to study pancreatic cancer; however, most studies do not provide data on the sensitivity and specificity of the developed biomarkers. Nevertheless, a multivariate prognostic model based on immune features (scRNA-seq) demonstrates an AUC of 0.901 for predicting 3-year survival and formed the basis of a nomogram for predicting overall survival [87].

CtDNA has emerged as a valuable prognostic tool. A study by D. Pietrasz et al. (2022) demonstrated that ctDNA status is an independent prognostic factor in metastatic pancreatic cancer: patients with ctDNA-positive results showed a median progression-free survival (PFS) of 5.3 months and overall survival (OS) of 8.2 months, whereas ctDNA-negative patients had longer survival—6.2 months PFS and 12.6 months OS [86]. Further research by M. Lin et al. (2018) focused on ctDNA carrying *KRAS* mutations and found it in 29.2% of samples. Here, the median OS was 11.4 months for ctDNA-positive patients versus 14.3 months for those with negative results, confirming the prognostic value of mutated ctDNA [17].

At the cellular level, single-cell RNA sequencing (scRNA-seq) has revealed important insights into tumor heterogeneity and immune interactions. K. Chen et al. (2023) used this technique to characterize immune profiles in pancreatic cancer tissue and adjacent non-cancerous tissues, ultimately developing a nomogram to predict patient survival [87]. Similarly, X. Zhao et al. (2021) examined ductal and stromal cells at the single-cell level, identifying *S100A2* as a potential biomarker for cancer cells [89]. The study also noted increased expression of *LGALS1*, *NPM1*, *RACK1*, and *PERP* in ductal cells, along with greater copy-number variation in ductal and cancer cells than in controls.

Comprehensive genomic analysis by S. Jones et al. (2008) uncovered 63 genetic alterations across 67–100% of tumours, affecting key signalling pathways that drive carcinogenesis [88]. This large-scale view was complemented by Y. Zhou et al. (2021), who applied scRNA-seq to metastatic pNET G2 samples and revealed intra- and inter-tumor heterogeneity in cell populations and transcriptional states [90]. The team developed a gene signature (*PCSK1* and *SMOC1*) to assess metastatic potential its prognostic value in a clinical cohort.

Specific molecular subtypes also show distinct clinical behaviors. A. Yavas et al. (2024) reported that SWI/SNF-deficient undifferentiated pancreatic carcinomas exhibit more aggressive disease progression [91]. Meanwhile, K. Tamura et al. (2018) identified defective variants in the *CPA1* and *CPB1* genes that are frequently found in patients with PDAC, implicating endoplasmic reticulum stress in disease development [92].

R. Bai et al. (2021) used bioinformatics analysis on the Gene Expression Profiling Interactive Analysis platform to identify lipid droplet-related genes as prognostic markers: 36 genes were upregulated and 3 downregulated in pancreatic cancer samples [93]. H.C. Bi et al. (2014) further linked cellular metabolism to tumor progression, showing that microRNA-1291 alters the metabolome of pancreatic cells and affects tumor development in mouse models [18]. Single-cell sequencing technology allows researchers to study every single cell within a tumour, offering unprecedented resolution in understanding tumour biology. This approach is set to advance further with the launch of large-scale initiatives such as the Human Cell Atlas project and the Human Biomolecular Atlas Program, which aim to dissect every tumour cell using genomic tools [94]. These projects promise to deepen our understanding of cellular diversity and interactions within the tumour microenvironment, paving the way for more precise diagnostic and therapeutic strategies.

Detection of *KRAS* mutations in pancreatic juice samples shows promise as a tool for differentiating pancreatic cancer from non-cancerous pancreatic diseases. These mutations may appear before clinical signs of cancer, suggesting their potential for early diagnosis. A proposed test uses *KRAS* mutation detection in pancreatic juice collected during endoscopic retrograde pancreatography, though further research is needed to establish its clinical significance [95].

Chronic pancreatitis poses a high risk of inflammation progressing to PDAC. Exploring the link between abnormally methylated extracellular DNA and precancerous lesions or chronic pancreatitis could revolutionize the development of early detection tools for PDAC [96]. This approach may enable identification of high-risk individuals at pre-malignant stages, allowing for timely intervention and improved outcomes.

Together, these findings highlight the diverse molecular and cellular landscape of pancreatic cancer and underscore the potential of ctDNA, single-cell profiling, and genomic analysis for improving diagnosis, prognosis, and personalized treatment strategies. The integration of emerging technologies and biomarkers holds particular promise for advancing early detection and risk stratification in this challenging disease.

Despite the considerable promise of multi-omics approaches in advancing pancreatic cancer research and diagnostics, several critical limitations hinder their rapid translation into clinical practice. One key challenge is the high costs and technical complexity of cutting-edge technologies, such as single-cell sequencing, high-resolution metabolomics, and deep metagenomic profiling. These factors limit accessibility and scalability in routine clinical settings. Another major issue is data heterogeneity and lack of standardization across studies. Differences in sample collection, processing protocols, sequencing platforms, and bioinformatic pipelines complicate data integration, comparison, and reproducibility. There is also a pressing need for extensive validation. Promising findings from discovery cohorts require confirmation in large, independent, and diverse populations to ensure generalizability and clinical utility. Integrating multi-omics datasets, including genomic, transcriptomic, metabolomic, and microbiome data, generates vast amounts of heterogeneous data. This demands advanced computational tools and specialized expertise for meaningful interpretation. Limited clinical interpretability presents another barrier. Even when molecular signatures are identified, translating them into actionable diagnostic or prognostic tools remains challenging. This is due to the complexity of biological networks and the need to account for confounding factors such as age, comorbidities, and lifestyle. Finally, regulatory and implementation barriers further delay clinical adoption. The path from research discovery to an FDA/EMA-approved diagnostic test involves rigorous clinical validation, quality control, and health-economic evaluation. Addressing these limitations through collaborative efforts, standardized protocols, and prospective clinical trials is essential to unlock the full potential of multi-omics in improving patient outcomes.

## 6. Conclusions

A comprehensive approach combining modern analytical methods, including ELISA, high-throughput sequencing, GC-MS, scRNA-seq, genomic analysis and metabolomics, is essential to improve the prognosis of pancreatic cancer. These techniques reveal the disease’s molecular, cellular and microbial heterogeneity, highlighting key changes in immune profiles, metabolic pathways and the tumor microbiome during progression from early lesions to metastasis.

While CA19-9 remains a widely used biomarker, its limited value for early diagnosis underscores the need to develop multi-marker prognostic models.

Understanding the interplay and the microbiome opens new opportunities for screening, early diagnosis, risk stratification, and treatment optimization. Integration of these data into clinical practice has the potential to significantly advance personalized pancreatic cancer treatment and improve patient outcomes, but further validation, standardization, and prospective clinical trials are needed to achieve this.

## Figures and Tables

**Table 1:** Diagnostic panels based on metabolomic profiles in serum pancreatic cancer patients.

No.	Key Metabolites/Markers	Methods of Analysis	AUC, Sensitivity/Specificity	Task and Results	Link
		Diagnostics			
1	Glucose, insulin	Immunohistochemistry; RNA sequencing	No data	Diagnosis with normal CA19-9. Additional assessment through metabolic indicators	[15]
2	206 metabolites, including palmitic acid, cholesterol, oleoyl-L-carnitine	LC-MS/MS	for palmitic acid and CA19-9, AUC = 1.000	Comprehensive metabolomic assessment. Significant differences between sick and healthy people	[12]
3	76 metabolites	LC-MS/MS, FIA-MS/MS	AUC = 0.9773	Differential diagnosis. High efficiency in distinguishing prostate cancer from other diseases	[33]
4	Ultra-long-chain fatty acids, phosphatidylcholines, sphingomyelins	High-resolution Fourier transform ion cyclotron resonance mass spectrometry (HR-FTICR-MS)	AUC = 0.97	Search for biomarkers. Detection of metabolic changes	[34]
5	Xylitol, 1,5-anhydro-D-glucitol, histidine, and inositol	GC-MS	1,5-Anhydo-D-glucitol: AUC = 0.83499, sensitivity 86.0%, specificity 71.4%	Diagnostics using metabolomics. Diagnostic model for pancreatic cancer	[26]
6	Serum heat shock protein 70 (HSP70)	A novel immunoelectrophoresis method developed and validated by the authors.	AUC = 0.7937	Early diagnosis. Significantly elevated in patients.	[35]
		Prognosis			
7	CA19-9, LRG1, TIMP1	ELISA analysis	An increment of 13.2% in sensitivity at 99% specificity (*p* = 0.031)	Early diagnosis. Increasing diagnostic sensitivity by combining markers.	[13]
8	CA19-9, metabolites PC.aa.C38_4, PC.ae.C42_5, PC.ae.C38_6	LC-MS/MS, FIA-MS/MS	AUC = 0.816	Predicting survival. Creation of a forecast nomogram.	[32]
9	Lymphocyte-to-leukocyte ratio (LNR)	Statistical data processing	No data	Significant prognostic factors for survival after pancreaticoduodenectomy for pancreatic adenocarcinoma.	[36]
10	Patients with resectable prostate cancer from the Surveillance, Epidemiology, and End Results database.	Statistical data processing	AUC = 0.845	A nomogram was developed to predict lymph node metastasis in patients with pancreatic cancer.	[37]
11	C-reactive protein	Statistical data processing	No data	The role of preoperative inflammatory response and postoperative pathological criteria based on C-reactive protein in identifying prognostic and/or predictive factors in pancreatic ductal adenocarcinoma.	[38]
12	Interleukin (IL)-6 and -8 levels	ELISA analysis	No data	Long-term low plasma IL-6/IL-8 levels as a prognostic marker for clinical outcomes of chemoimmunotherapy.	[39]
13	Serum cytokines: expression of B7-1/CD80, EG-VEGF/PK1, IL-29, NRG1-beta1/HRG1-beta1, and PD-ECGF	ELISA analysis	Average goodness of fit (R-squared measurement) of 0.914, accuracy 92.3%, sensitivity 85.57%, specificity 100%	A multivariate model showed superior results and may represent a new panel of serum cytokines that correlate with poor prognosis.	[40]
14	Neutrophil-to-lymphocyte ratio	Statistical data processing	No data	The prognostic value of the neutrophil-to-lymphocyte ratio (NLR) and platelet-to-lymphocyte ratio (PLR) for survival rates and the risk of metastasis in prostate cancer.	[41]
		Research			
15	N-succinyl-L-diaminopimelic acid, phosphatidylethanolamine	HPLC	AUC = 0.951, sensitivity 93.3%, specificity 93.1%	Pancreatic cancer screening in the presence of diabetes. Two-metabolite panel with high sensitivity (93.3%) and specificity (93.1%).	[42]
16	CA19-9, HbA1c	HPLC	AUC = 0.81, sensitivity 70.0%, specificity 96.5%	CA19-9 assessment in diabetes. Correlation of CA19-9 with glycated hemoglobin.	[14]

**Table 2:** Microbiome profiles as potential biomarkers for pancreatic cancer.

No.	Method, Object	AUC, Sensitivity/Specificity	Microbiome Changes Detected	Link
		Intestinal microbiota		
1	Fecal sample/intratumoral microbiome screening; metagenomic and 16S rRNA sequencing	AUROC 0.84 for PDAC detection; 0.94 when combined with CA19-9	Taxa enriched in fecal samples: *Veillonella*, *Streptococcus, Akkermansia*; taxa with different abundance in healthy and tumor pancreatic tissues: *Bacteroides*, *Lactobacillus*, *Bifidobacterium*. AUROC 0.84 for PDAC detection; 0.94 when combined with CA19-9	[58]
2	Fecal samples; 16S rDNA sequencing	No data	The differences in species composition in the goal-directed intravenous therapy group versus the liberal intravenous fluid infusion group; more bacteria involved in metabolism and restoration of blood coagulation function, including *Prevotella*, *Roseburia*, *Lachnospiracea*, *Dialister*, and *Clostridium*; the liberal intravenous fluid infusion group contained more opportunistic pathogens, including *Enterococcus*, *Pseudomonas aeruginosa*, and *Acinetobacter baumannii*.	[59]
3	Fecal samples; 16S rRNA sequencing	No data	*Proteobacteria*, *Actinobacteria*, *Fusobacteria* and *Verrucomicrobia* were higher in the gut of patients with PDAC compared to healthy controls. Interestingly, *Proteobacteria* were also enriched in the intrapancreatic microbiome of patients with PDAC and were associated with disease progression.	[60]
4	The intestinal microbiota; 16S rRNA sequencing	AUC 0.842 based on 40 genera associated with PDAC	Dominant phyla: *Bacteroidetes*, *Firmicutes*, *Proteobacteria*. In cancer patients: ↑ *Bacteroidetes*, ↓ *Firmicutes* and *Proteobacteria.* At the genus level: enriched *Prevotella*, *Veillonella*, *Klebsiella*, etc.; decreased *Gemmiger*, *Bifidobacterium*, *Coprococcus*, *Clostridium IV*, *Blautia*, *Flavonifractor*, *Anaerostipes*, *Butyricicoccus*, and *Dorea*	[61]
5	Secretin-stimulated duodenal fluid; 16S and 18S rRNA sequencing	No data	Enrichment of duodenal fluid with the genus *Bifidobacterium* (compared to the control group and patients with pancreatic cysts). In patients with short-term survival from pancreatic cancer, enrichment of *Fusobacteria* and *Rothia* was found.	[62]
6	Duodenal microbiome; 16S rRNA gene pyrosequencing Additionally: endotoxin analysis, IL-6, CRP, *H. pylori* test, biopsy histology	No data	In patients with cancer, the duodenal mucosa is enriched with *Acinetobacter*, *Aquabacterium*, *Oceanobacillus*, *Rahnella*, *Massilia*, *Delftia*, *Deinococcus* and *Sphingobium*. In healthy individuals, it is enriched with *Porphyromonas*, *Paenibacillus*, *Enhydrobacter*, *Escherichia*, *Shigella* and *Pseudomonas.*	[63]
		Other types of microbiotas		
7	The saliva microbiota; quantitative real-time PCR (qPCR).	For pancreatic cancer versus healthy control, the combination of two microbial biomarkers (N elongata and S mitis) yielded a ROC-plot AUC value of 0.90 (95% CI 0.78 to 0.96, *p* < 0.0001) with 96.4% sensitivity and 82.1% specificity	In patients with pancreatic cancer, 31 species/clusters were elevated, and 25 were decreased compared to healthy controls. Dominant groups: *Firmicutes* (*Streptococcus*, *Granulicatella*), *Proteobacteria* (*Campylobacter*, *Neisseria*), CFB group (*Prevotella*, *Porphyromonas*), *Actinobacteria* (*Atopobium*, *Rothia*)	[23]
8	The tongue plaque microbiota; 16S rRNA sequencing	Fusobacterium, Leptotrichia and Porphyromonas, as microbial indexes, AUC 0.802, sensitivity 0.771, and 0.786 for specificity	Low levels of *Haemophilus* and *Porphyromonas*, high levels of *Leptotrichia* and *Fusobacterium* in patients with primary pancreatic cancer compared with healthy controls	[24]
9	Oral wash samples; 16S rRNA sequencing	No data	The oral pathogens *Porphyromonas gingivalis* and *Aggregatibacter actinomycetemcomitans* were associated with an increased risk of PDAC. *Fusobacteria* and their genus *Leptotrichia* were associated with a reduced risk of PDAC.	[64]
10	Intratumor microbiome compared with normal adjacent tissue (NAT)	No data	Microbial α-diversity was decreased in PDAC tumors. The relative abundance of *Staphylococcus aureus*, *Cutibacterium acnes*, and *Cutibacterium granulosum* was higher in pancreatic tumors, and the presence of *Ralstonia pickettii_B* was associated with worse overall survival. Metabolomic analysis revealed significant compositional differences between pancreatic tumors and normal pancreatic tissue, with 553 discriminant metabolites identified. The differential metabolites originated from the microbiota and demonstrated significant interactions with biased bacterial species through KO genes (KEGG Orthology). The pancreatic tumor microenvironment contains unique enzymatic reactions of microbial origin that potentially influence pancreatic tumor initiation and progression by modulating glycerol-3-phosphate, succinate, carbonate, and beta-alanine levels	[19]
11	Tumor tissue; 16S rRNA sequencing	Top 3 taxa (*Pseudoxanthomonas*, *Saccharopolyspora* and *Streptomyces*) AUC 88.89; with *Bacilus Clausii* up to AUC 97.51	To evaluate the prognostic significance of the tumor microbiome for long-term survival patients. Higher alpha diversity was found in the tumor microbiome of patients with long-term survival. An intratumor microbial signature profile (*Pseudoxanthomonas/Streptomyces/Saccharopolyspora/Bacillus clausii*) was identified that significantly predicted long-term survival in the research and validation cohorts. Predicts long term survival; associated with CD8+ T cell infiltration	[65]
12	PDAC and adjacent normal tissues; 16S rDNA sequencing, metabolomic profiling by LC–MS/MS analysis	No data	(α-diversity and β-diversity) of the intratumor microbiome, 298 metabolites that were significantly altered in pancreatic cancer, including glycine, serine, and threonine metabolism, amino acid biosynthesis, and metabolic pathways. *Pseudomonas* was the predominant species in PDAC.	[10]
13	The PDAC tissues; 16S rRNA amplicon sequencing and untargeted metabolomics by LC/MS	No data	A pattern of microbial dysbiosis and metabolic changes, with DCs representing a transitional state in cancer development. Furthermore, glycoprotein A and S-(2-carboxyethyl)-L-cysteine are associated with tumor progression and chemotherapy resistance. In PDAC cases, an increase in the abundance of *Peptostreptococcus*, *norank_f__Mitochondria*, and *Ileibacterium* was observed, while the abundance of *Faecalibacterium*, *Ruminococcus*, *Subdoligranulum*, and *Coprococcus* was consistently decreased. Among the altered metabolites, beta-guanidinopropionic acid, S-(2-carboxyethyl)-L-cysteine, L-lactic acid, and 3-hydroxybutanoic acid increased, while (3Z)-2-propylpent-3-enoic acid, linoleic acid, and monopalmitin consistently decreased	[11]

**Table 3:** Studies focusing on metagenomic changes occurring directly in pancreatic tissues, reflecting shifts in tumor cells and healthy tissues, and the possibility of diagnosis or prognosis based on these changes.

No.	Object of Study	Methods of Analysis	Task and Results	Link
1	Blood serum; circulating tumor DNA (PCR) and next-generation sequencing (NGS)	To determine whether ctDNA is an independent prognostic factor in mAPh.	Median progression-free survival (PFS) and overall survival (OS) were 5.3 and 8.2 months in ctDNA-positive patients and 6.2 and 12.6 months in ctDNA-negative patients, respectively.	[86]
2	Blood serum; Droplet digital polymerase chain reaction (ddPCR)	The aim of the study was to evaluate whether circulating tumor DNA (ctDNA) with mutated *KRAS* genes is a prognostic factor for survival in patients with pancreatic cancer.	Circulating tumor DNA (ctDNA) was detected in 20 (29.2%) samples. The median overall survival (OS) was 11.4 months in patients with a ctDNA-positive result and 14.3 months in patients with a ctDNA-negative result.	[17]
3	The pancreatic cancer tissue and adjacent non-cancerous tissues.	Single-cell RNA sequencing (scRNA-seq)	To further characterize pancreatic cancer immune profiles and study cell–cell interactions. A nomogram to predict overall patient survival.	[87]
4	Tumor DNA.	Next-generation sequencing technologies (NGS)	To identify additional genes and signaling pathways. A total of 63 genetic alterations in 67–100% of tumors.	[88]
5	Ductal and stromal cells	Single-cell RNA sequencing (scRNA-seq). were examined from an individual cell perspective.	Changes in ductal and stromal cells during PASC progression. S100A2 to be a potential biomarker for cancer cells. Expression of LGALS1, NPM1, RACK1, and PERP was increased in ductal cells. Copy number variations were greater in ductal cells and cancer cells than in control cells. Expression of EREG, FCGR2A, CCL4L2, and CTSC increased in myeloid cells.	[89]
6	The tumor tissue samples, samples of normal liver tissue, peripheral blood mononuclear cells from patients with metastatic pNET G2.	Single-cell RNA sequencing (scRNA-seq).	ScRNA-seq analysis revealed intra- and intertumor heterogeneity in cell populations, transcriptional states, and cell–cell interactions. A gene signature (*PCSK1* and *SMOC1*) was developed to determine tumor metastatic potential, and its prognostic value was validated	[90]
7	A screening of switch/sucrose non-fermentable (SWI/SNF) chromatin remodeling complexes -deficient undifferentiated pancreatic carcinomas.	Immunohistochemistry (IHC) and next-generation sequencing (NGS).	To further understand SWI/SNF-deficient undifferentiated pancreatic carcinomas. SWI/SNF-deficient undifferentiated pancreatic carcinomas have a more aggressive behavior.	[91]
8	Pancreatic acinar cells	Next-generation sequencing (NGS).	Defective variants in the *CPA1* and *CPB1* genes are more common in patients with pancreatic cancer.	[92]
9	Pancreatic cancer and normal pancreatic tissue samples.	Bioinformatics analysis on the GEPIA platform (data are publicly available).	To identify lipid droplet-related genes as prognostic markers in pancreatic cancer. In total, 36 genes were upregulated and 3 genes were downregulated	[93]
10	Tissue specimens	Quantitative real-time PCR (qPCR) analysis and Ultra-High-Performance Liquid Chromatography–Electrospray Ionization–Quadrupole Time-of-Flight Mass Spectrometry analysis	Changes in the pancreatic cell metabolome under the influence of microRNA-1291 and tumor development in mouse models.	[18]

Abbreviations: *KRAS*—Kirsten rat sarcoma viral oncogene homolog; S100A2—S100 calcium-binding protein A2; LGALS1—galectin-1; NPM1—nucleophosmin 1; RACK1—receptor for activated C kinase 1; PERP—p53 apoptosis effector related to PMP-22; EREG—epiregulin; FCGR2A—Fc gamma receptor IIa; CCL4L2—C-C motif chemokine ligand 4 like 2; CTSC—cathepsin C; *PCSK1*—proprotein convertase subtilisin/kexin type 1; *SMOC1*—SPARC related modular calcium binding 1; *CPA1*—carboxypeptidase A1; *CPB1*—carboxypeptidase B.

## Data Availability

No new data were created or analyzed in this study. Data sharing is not applicable to this article.

## Abbreviations

The following abbreviations are used in this manuscript:

PDAC	Pancreatic cancer/Pancreatic ductal adenocarcinoma
ELISA	Enzyme-linked immunosorbent assay
CA19-9	Carbohydrate antigen 19-9
PCR	Polymerase chain reaction
scRNA-seq	RNA sequencing
LPS	Lipopolysaccharide
HbA1c	Glycated hemoglobin
HSP70	Heat shock protein 70
GC-MS	Gas chromatography–mass spectrometry
HPLC	High-performance liquid chromatography
LC-MS/MS	Liquid chromatography with tandem mass spectrometry
FIA-MS/MS	Flow injection analysis with tandem mass spectrometry
IL-6/IL-8	Interleukin-6/interleukin-8
LRG1	Leucine-rich alpha-2-glycoprotein 1
TIMP1	Tissue inhibitor of metalloproteinases-1
NLR	Neutrophil-to-lymphocyte ratio
BCAA	Branched-chain amino acid
DNA	Deoxyribonucleic acid
mtDNA	Mitochondrial DNA
ctDNA	Circulating tumour DNA
TCA cycle	Tricarboxylic acid cycle
CRP	C-reactive protein
qPCR	Real-time PCR
SCFAs	Short-chain fatty acids
TMAO	Trimethylamine N-oxide
TMA	trimethylamine
3-IAA	Indole-3-acetic acid
AhR	Aryl hydrocarbon receptor
NGS	Next-generation sequencing
*H. pylori*	*Helicobacter pylori*
IDO1	Indoleamine 2,3-dioxygenase 1
OS	Overall survival
